# Hypoxia imaging and theranostic potential of [^64^Cu][Cu(ATSM)] and ionic Cu(II) salts: a review of current evidence and discussion of the retention mechanisms

**DOI:** 10.1186/s13550-020-00621-5

**Published:** 2020-04-09

**Authors:** Tengzhi Liu, Morten Karlsen, Anna Maria Karlberg, Kathrine Røe Redalen

**Affiliations:** 1 Department of Physics, Norwegian University of Science and Technology, Høgskoleringen 5, 7491 Trondheim, Norway; 2 Department of Radiology and Nuclear Medicine, St. Olavs hospital, Trondheim University Hospital, Trondheim, Norway; 3 Department of Circulation and Medical Imaging, Norwegian University of Science and Technology, Trondheim, Norway

**Keywords:** Copper-64, Metabolism, Positron emission tomography, Tumor hypoxia, Radiation

## Abstract

**Background:**

Tumor hypoxia (low tissue oxygenation) is an adverse condition of the solid tumor environment, associated with malignant progression, radiotherapy resistance, and poor prognosis. One method to detect tumor hypoxia is by positron emission tomography (PET) with the tracer [^64^Cu][Cu-diacetyl-bis(N(4)-methylthiosemicarbazone)] ([^64^Cu][Cu(ATSM)]), as demonstrated in both preclinical and clinical studies. In addition, emerging studies suggest using [^64^Cu][Cu(ATSM)] for molecular radiotherapy, mainly due to the release of therapeutic Auger electrons from copper-64, making [^64^Cu][Cu(ATSM)] a “theranostic” agent. However, the radiocopper retention based on a metal-ligand dissociation mechanism under hypoxia has long been controversial. Recent studies using ionic Cu(II) salts as tracers have raised further questions on the original mechanism and proposed a potential role of copper itself in the tracer uptake. We have reviewed the evidence of using the copper radiopharmaceuticals [^60/61/62/64^Cu][Cu(ATSM)]/ionic copper salts for PET imaging of tumor hypoxia, their possible therapeutic applications, issues related to the metal-ligand dissociation mechanism, and possible explanations of copper trapping based on studies of the copper metabolism under hypoxia.

**Results:**

We found that hypoxia selectivity of [^64^Cu][Cu(ATSM)] has been clearly demonstrated in both preclinical and clinical studies. Preclinical therapeutic studies in mice have also demonstrated promising results, recently reporting significant tumor volume reductions and improved survival in a dose-dependent manner. Cu(II)-[Cu(ATSM)] appears to be accumulated in regions with substantially higher CD133^+^ expression, a marker for cancer stem cells. This, combined with the reported requirement of copper for activation of the hypoxia inducible factor 1 (HIF-1), provides a possible explanation for the therapeutic effects of [^64^Cu][Cu(ATSM)]. Comparisons between [^64^Cu][Cu(ATSM)] and ionic Cu(II) salts have showed similar results in both imaging and therapeutic studies, supporting the argument for the central role of copper itself in the retention mechanism.

**Conclusions:**

We found promising evidence of using copper-64 radiopharmaceuticals for both PET imaging and treatment of hypoxic tumors. The Cu(II)-[Cu(ATSM)] retention mechanism remains controversial and future mechanistic studies should be focused on understanding the role of copper itself in the hypoxic tumor metabolism.

## Background

Tumor hypoxia is an adverse condition of the tumor microenvironment defined by low tissue oxygenation as a result of an imbalance between cellular oxygen supply and consumption [1]. The cause of tumor hypoxia includes poor perfusion due to lack of blood flow, inadequate oxygen diffusion due to increased distance with tumor expansion, or reduced transport capacity of oxygen in blood (anemia) [2]. Tumor hypoxia is typically associated with malignant progression, metastasis, resistance to chemo- and/or radiotherapy, recurrence, and overall poor prognosis [3–5]. Tumor hypoxia can also induce aggravating biological changes in the tumor microenvironment that result in change of gene expression patterns, alter the malignant potential of the tumor, and lead to increased tumor aggressiveness [6]. Detection of tumor hypoxia is therefore of great importance in order to optimize the treatment strategy and improve overall prognosis [2, 7].

Conventionally, assessment of tumor hypoxia has been achieved by using invasive techniques such as measuring the oxygen partial pressure (pO_2_) with intratumoral polarographic sensors (an oxygen probe), developed in the 1990s [3, 8]. Although invasive methods such as polarographic electrodes have the advantage of high sensitivity and are often regarded as gold standards, these methods also suffer from limited tumor accessibility, disruption of the tumor microenvironment, and difficulties to distinguish necrotic regions of the tumor, in addition to the troublesome and painful procedures [9]. As a result, using invasive techniques has questionable oxygen level representation of the tumor as a whole as well as difficulties to visualize variations of oxygen concentration within the tumor [5]. A number of noninvasive techniques can be used to image tumor hypoxia, including positron emission tomography (PET), functional magnetic resonance imaging (fMRI), electron paramagnetic resonance (EPR), and optical spectroscopy. Though based on different principles, these techniques share similar advantages that allow serial tracking of hypoxic regions and accessibility that are otherwise challenging for invasive techniques [5]. In blood oxygen level dependent (BOLD) fMRI, the detection of hypoxia is based on an indirect mechanism by measuring the magnetic susceptibility difference between deoxy-hemoglobin (paramagnetic) and oxy-hemoglobin (diamagnetic). The measurement is influenced by for instance the change of hemoglobin concentration and interconversions, thus the result is considered as qualitative assessments of oxygenation dynamics but not direct oxygen levels [10]. In contrast, detection of tumor hypoxia in PET is a direct measurement, presenting higher sensitivity and is directly quantitative. Compared to the more commonly used fluorine-based PET tracers, tracers based on copper allow a rapid visualization of hypoxia (in contrast to [^18^F]F-MISO), or better penetration of the blood-brain barrier and no urinary bladder uptake (in contrast to [^18^F]FAZA) [11, 12], in addition to longer half-lives that contributes to better image quality and more convenient clinical practice. Here, we focus on reviewing recent advances and challenges in imaging of tumor hypoxia using PET with one of the most promising copper tracers, [^64^Cu][Cu-diacetyl-bis(N^4^-methylthiosemicarbazone)] ([^64^Cu][Cu(ATSM)]), and simple ionic [^64^Cu]Cu(II) salts, as well as implications on using these tracers for simultaneous internal molecular radiotherapy.

## Results

### Copper-labelled ATSM

There has been a number of preclinical studies on radiolabeled copper [Cu(ATSM)] (may be abbreviated as [^*^Cu][Cu(ATSM)] when positron-emitting radiocopper is used and the choice of isotope does not have impact on the chemical properties of the tracers) as PET tracers for the imaging of tumor hypoxia in varies tumor types. To highlight a few, the first reported study by Fujibayashi et al. in 1997 used [^62^Cu][Cu(ATSM)] for detection of hypoxia, where a perfused rat heart model was imaged and sustained accumulation of [^62^Cu][Cu(ATSM)] was observed under hypoxia, but not normoxia or reoxygenation [13]. Selectivity of copper-labelled ATSM in hypoxic tumor regions has since been explored. In a comparative in vivo and in vitro study between [^64^Cu][Cu(ATSM)], [^64^Cu][Cu-pyruvaldehyde-bis(N4-methylthiosemicarbazone)] ([^64^Cu][Cu(PTSM)]), and [^18^F]-fluoromisonidazole ([^18^F]-FMISO), Lewis et al. demonstrated in a hypoxic EMT6 breast cancer model that uptake of [^64^Cu][Cu(ATSM)] was oxygen concentration dependent, and compared to [^18^F]-FMISO, [^64^Cu][Cu(ATSM)] had higher hypoxia cellular uptakes and more rapid washout in normoxic cells. While [^64^Cu][Cu(PTSM)] had a uniform distribution throughout the tumor, [^64^Cu][Cu(ATSM)] showed heterogeneity in distribution within the tumor, suggesting selective trapping in hypoxic cells [14]. Later, the same group confirmed with polarographic oxygen electrodes that both the [^61^Cu]-, [^64^Cu]-, and [^67^Cu][Cu(ATSM)] retention imaged with PET and with electronic autoradiography was pO_2_ dependent [15]. Tanaka et al. compared the regional distribution of [^64^Cu][Cu(ATSM)] accumulated inside the tumor mass in four different experimental tumor models (LLC-1, Meth-A, B16, and colon-26) with immunohistochemical staining and demonstrated hypovascular and cell-cycle-arrest featured in these regions [16]. Vāvere et al. compared [^64^Cu][Cu(ATSM)] with [^18^F]-FMISO for PET imaging of tumor hypoxia and revealed that [^64^Cu][Cu(ATSM)] was advantageous in terms of more rapid kinetics, better hypoxia to background ratio, and higher detection threshold [17].

Further comparisons of the intratumoral distribution between [^64^Cu][Cu(ATSM)] and the most commonly used PET tracer, ^18^F-fluorodeoxyglucose ([^18^F]-FDG), by Obata et al. in an VX2 carcinoma xenograft rabbit model revealed some interesting insights. It was found that [^64^Cu][Cu(ATSM)] was mainly accumulated in the outer rim of the tumor, thought to be the most aggressive part of the tumor, with clusters of viable tumor cells and under hypoxia due to active proliferation, whereas [^18^F]-FDG accumulated in the regions of pre-necrotic cells or cells with irreversible damages [18]. Oh et al. further revealed that tumor cells in high [^64^Cu][Cu(ATSM)] accumulation regions are quiescent but highly clonogenic and are rapidly proliferating, induced by mild hypoxia and under a highly reductive environment, consistent with prior studies [18, 19]. Using a mouse colon carcinoma model, Yoshii et al. discovered that [^64^Cu][Cu(ATSM)] indeed preferentially accumulates in regions of tumors with high expression of CD133+, which are characterized as cancer stem cells (CSCs) and enriched under hypoxia due to survival advantages, as well as promoted self-renewal ability through the activation of the hypoxia inducible factor-1α (HIF-1α) [20–22]. In addition, it has also been suggested that [^64^Cu][Cu(ATSM)] is a direct indicator of an over-reduced intracellular state caused by hypoxia, and thus, an indirect indicator of hypoxia [23]. Collectively, these preclinical studies have provided evidence to suggest the use of radiolabeled copper [Cu(ATSM)] not only as a tracer for imaging of tumor hypoxia, but potentially as a tracer of clonogenic cancer stem cells or stem-like cells which accumulate in regions characterized by a highly reductive biochemical environment.

### Ionic copper-64

Using simple [^64^Cu]Cu-dichloride ([^64^Cu]CuCl_2_), Peng et al. were able to visualize mouse hepatoma xenografts with small animal PET, where the copper uptake is believed to be mediated by the copper transporter 1 (CTR-1) [24]. Later, the same group observed increased tumor uptake of copper-64 in a human prostate cancer xenograft mouse model and concluded that [^64^Cu]CuCl_2_ PET may be useful for the detection of local recurrence in the prostate bed [25]. Similarly, Zhang et al. later visualized human hepatocellular carcinoma in a xenograft model using [^64^Cu]CuCl_2_ as the PET tracer [26]. Concerning the stability of ligand-bound copper-64 tracers from other studies, Jørgensen et al. investigated the uptake of copper-64 from [^64^Cu]CuCl_2_ in five different xenograft models (from colorectal cancer, glioblastoma, head and neck cancer, neuroendocrine lung carcinoma, and ovarian cancer) and found high tumor uptake of copper-64 in four out of the five models, with the exception of ovarian cancer having moderate uptake [27].

To investigate the role of copper itself in the retention mechanism, Hueting et al. intriguingly discovered in an in vivo study that when comparing the behavior of [^64^Cu]Cu-acetate and [^64^Cu][Cu(ATSM)] in EMT6 and HT1080 mouse xenograft models, the uptake and distribution of copper-64 showed mirroring performance of the two tracers [28]. Furthermore, when increasing the inhalation of O_2_ at two hours retention, the uptake of both tracers was also correspondingly reduced [28]. Contrary to the in vivo results, in vitro experiments showed substantially less uptake of [^64^Cu]Cu-acetate than [^64^Cu][Cu(ATSM)]. In addition, radio thin layer chromatography (TLC) showed that although [^64^Cu][Cu(ATSM)] appeared to be stable in mouse serum in vitro, ex vivo analysis of mouse serum extracted from blood injected with [^64^Cu][Cu(ATSM)] showed that copper exists primarily in the form of ionic [^64^Cu]Cu(II) with comparable amount of copper-64 radioactivity as in a mouse injected with [^64^Cu]Cu-acetate [28]. Using an octanol-extraction method to analyze the amount of intact [^64^Cu][Cu(ATSM)] in mouse whole blood, the same group reported that only 3% of the copper complex remained intact 30 min after injection, while the rest existed as serum-bound copper-64, having radioactivity comparable to the extraction from mice injected with [^64^Cu]Cu-acetate [28]. In summary, the results from these authors seem to question the validity of the previously proposed copper retention mechanism based on the instability of [^64^Cu][Cu(ATSM)] complex in vivo [28]. Such instability will lead to the dismantling of [^64^Cu]Cu(II) from the complex before reaching the tumor cells, despite the mechanism has been demonstrated in vitro [28]. Ferrari et al. further investigated the theranostic possibility of [^64^Cu]CuCl_2_ in a glioblastoma xenograft model and found good visualization of tumors, and noted that the ability of copper ions to enter cancer cells may not simply depend on the complexing ligand, but rather be tightly linked to copper itself [29].

### Clinical studies with [^*^Cu][Cu(ATSM)] and ionic copper-64

The possibility of using radiolabeled copper [Cu(ATSM)] and ionic Cu(II) salts for PET imaging of tumor has also been demonstrated in clinical studies with different cancer types, including lung, cervical, rectal, head and neck, brain, and prostate cancer. One of the reasons these cancer types were chosen in the studies with [^*^Cu][Cu(ATSM)] PET imaging is because of the difficulties to detect hypoxia due to the physical inaccessibility to use invasive methods [30–33]. Although the exact mechanism and relations to tissue hypoxia explaining the retention of either [^64^Cu][Cu(ATSM)] or ionic copper-64 is yet to be identified, the benefits of using these PET tracers have been demonstrated clinically. For instance, Dietz et al. demonstrated the effectiveness of [^60^Cu][Cu(ATSM)] as a predictor of neoadjuvant chemoradiotherapy response and survival of patients with rectal cancer [34]. More recently, it has been reported that radiolabeled copper [^*^Cu][Cu(ATSM)]/[^*^Cu]CuCl_2_ can be used as a predictor of radiotherapy response in head and neck cancer ([^62^Cu][Cu(ATSM)]) [35], staging and detection of recurrent prostate cancer ([^64^Cu]CuCl_2_) [36, 37], and imaging of brain tumors ([^64^Cu]CuCl_2_) [38]. A summary of clinical studies with either copper-labelled ATSM or ionic radiocopper is shown in Table 1.

**Table 1:** Clinical studies using *copper(II)-diacetyl-bis(N4-methylthiosemicarbazone) ([^*^Cu][Cu(ATSM)]) or *Cu-dichloride ([^*^Cu]CuCl_2_) as radioactive tracer in PET

Tracer	Tumor type	Patient No.	Results/Conclusions	Reference
[*Cu][Cu(ATSM)]	Lung cancer	6	^62^Cu-ATSM rapidly accumulated in tumors of patients with lung cancer but not in the lung of healthy volunteers. The level of radiocopper reached abnormally intense plateau levels within a few minutes after intravenous administration in tumors with different distribution from [^18^F]-FDG.	[39]
	Lung cancer (NSCLC)	19	PET imaging with ^60^Cu-ATSM in patients with NCSLC is feasible. At a tumor (T)/muscle (M) threshold of 3.0 clear distinct prediction can be made between patients that respond to therapy (mean T/M = 1.5 ± 0.4) and the non-responders (mean T/M = 3.4 ± 0.8). No significant difference in tumor SUV was observed with [^18^F]-FDG.	[31]
	Cervical cancer	14	Tumor uptake of ^60^Cu-ATSM inversely correlate with progression-free and overall survival. Uptake of [^18^F]-FDG did not correlate with ^60^Cu-ATSM and no significant [^18^F]-FDG distribution difference between hypoxic and normoxic tumors.	[40]
	Cervical cancer	15	Patients with hypoxic cancer as determined by ^60^Cu-ATSM correlates with overexpressed VEGF, EGFR, COX-2, CA-9, as well as high apoptotic and worse prognosis.	[110]
	Cervical cancer	38	Patients were monitored through a period from 3 to 79 months. With a threshold of T/M = 3.5 the 3-year progression free and cause-specific survival rate of patients with normoxic tumors (T/M < 3.5, 71%) is significantly higher than that of hypoxic tumor (T/M > 3.5, 28%). Advantages of ^60^Cu-ATSM over ^18^F-FMISO include much faster pharmacokinetics which results in better target-to-background ratio, and no interference due to urinary bladder uptake.	[32]
	Rectal	19	^60^Cu-ATSM PET may be predictive of survival and possible tumor response to neoadjuvant chemoradiotherapy in rectal cancer.	[34]
	Cervical cancer	10	PET images from [^64^Cu][Cu(ATSM)] have better image quality (lower noise) than images from [^60^Cu][Cu(ATSM)]. Patterns and magnitude of the tumors were similar between the two tracers with different copper isotopes.	[111]
	Lung cancer	13	SUV of ^62^Cu-ATSM is higher than [^18^F]-FDG at the peripheral region of tumor while [^18^F]-FDG is higher at the center of the tumor. This difference of intratumor distribution indicates the difference of high glucose metabolism regions and hypoxia region inside various histopathologic types of lung cancer.	[41]
	Head and neck	17	Increased uptake of [^62^Cu][Cu(ATSM)] predicted tumor resistance to chemo- and radiotherapy as well as recurrence.	[35]
	Head and neck	30	Among 27 patients with squamous cell carcinoma, [^62^Cu][Cu(ATSM)] showed higher accumulation in the peripheral regions than in the center of the tumor mass, opposite to the accumulation pattern of [^18^F]-FDG. In 3 patients with adenocarcinoma, the accumulation of both ^62^Cu-ATSM and [^18^F]-FDG were homogeneous.	[33]
	Glioma	22	Uptake (SUV_max_) and T/B ratio of [^62^Cu][Cu(ATSM)] were much higher in grade IV than grade III/II gliomas, correlated with MRI findings of necrosis. At T/B=1.8 threshold, [^62^Cu][Cu(ATSM)] is predictive of HIF-1a expression, with 92.3% sensitivity and 88.9% specificity.	[112]
	Head and neck	25	At T/M = 3.2 [^62^Cu][Cu(ATSM)] uptake threshold, patients with hypoxic tumors have significant worse 2-year progression free survival (20%) and cause-specific survival (33%), compares to patients with lower [^62^Cu][Cu(ATSM)] uptake (73% and 80%, respectively).	[113]
	Head and neck	11	[^64^Cu][Cu(ATSM)] has very high sensitivity (100%) of predicting radiotherapy response, but relatively low specificity for the prediction of neoadjuvant chemoradiotherapy response.	[114]
	Head and neck	30	With a cut-off TMR of 3.2, patients with hypoxic tumors have significantly worse progression-free survival and cause-specific survival. ^62^Cu-ATSM is predictive of treatment resistance and poor prognosis for head and neck cancers.	[115]
	Lung /H&N	7/11	Moderate to high uptake of [^64^Cu][Cu(ATSM)] were found in all patients. No statistic significant difference in SUV between the two tumor types.	[116]
Ionic copper (II)	Prostate cancer	7	Due to the absence of urinary excretion lesions in the pelvic area can be easily imaged with [^64^Cu]CuCl_2_-PET. No adverse or clinical detectable pharmacologic effects were observed in any of the patients at mean activity of 339 MBq (4 MBq/kg).	[36]
	Brain cancer	19	Using [^64^Cu]CuCl_2_ the brain cancerous lesions can be clearly visualized at 1 h after the injection and have stable retention between 3-24 hours. Accumulation of the tracer in consistent agreement with MRI images. The brain lesions were also clearly visualized in patients simultaneously affected by other types of cancer.	[38]
	Healthy volunteer	6	Healthy volunteers were injected with [^64^Cu]CuCl_2_ at a dose of 4.0 MBq/kg. No observable adverse effects, clinically detectable pharmacologic effects, and change in standard vital signs were observed. Uptake of copper-64 was observed predominantly in liver, followed by bowel and kidneys.	[101]
	Prostate cancer	50	Among the 50 patients, 41 were detected (82%) positive via [^64^Cu]Cu-PET, compared to 56% using [^18^F]F-choline PET and 74% using multiparametric MRI. Unlike [^18^F]F-choline, [^64^Cu]CuCl_2_ allows thorough pelvic exploration due to lack of excreted or accumulated urinary tract.	[37]
	Prostate cancer	50	The maximum tumor-to-background ratio was reached 1 hour after injection. Mean SUV_max_ in lymph nodes and bone metastases were significantly higher than local relapse. Discounting the Auger electrons, the mean absorbed dose for PCa lesions per administration was ~0.06 mGy/MBq. The therapeutic effect of [^64^Cu]CuCl_2_ is mainly dependent on the Auger electron emission instead of beta radiation.	[58]

* Atomic mass number

These clinical studies have demonstrated the possibility of using radiolabeled copper [^*^Cu][Cu(ATSM)]/[^*^Cu]CuCl_2_ for PET imaging of solid tumors and potentially for hypoxia imaging. In addition, therapy response and overall survival may be possible to predict with the aid of tissue-to-muscle ratios. Compared to [^18^F]-FDG, radiolabeled copper [^*^Cu][Cu(ATSM)] have been able to selectively image heterogeneity in tumor hypoxia within the tumor mass [31, 33, 39–41]. Compared to fluorine-based hypoxic PET tracers such as [^18^F]-FMISO and [^18^F]-choline, radiolabeled copper [^*^Cu][Cu(ATSM)]/[^*^Cu]CuCl_2_ have the advantages of much faster pharmacokinetics, better signal-to-noise ratio, and no interference due to absence of urinary bladder uptake [36, 37, 42]. Furthermore, ionic [^64^Cu]Cu^2+^ have also demonstrated improved diagnostic sensitivity in a recent study involving 50 patients with prostate cancer, with 82% detection rate compared to 74% using multiparametric MRI, which is the standard radiologic procedure, and 56% using [^18^F]-choline-PET, although the authors did not report comparisons with the more recently developed prostate-specific membrane antigen (PSMA)-based PET tracer [37]. Despite early experiments were mainly based on radiolabeled copper [^*^Cu][Cu(ATSM)], later studies have also involved ionic radiocopper and reached similar results. Notably, if the copper retention indeed followed solely the ligand-based trapping mechanism, using ionic radiocopper should not cause any selective accumulation in hypoxic tumors. Combining the clinical and in vivo preclinical results, it is clear that copper itself also plays an important role in the hypoxic selectivity and therefore should be emphasized.

### Role of Cu-64 in therapeutic applications

Recently, several preclinical studies have reported the possibility of using [^64^Cu][Cu(ATSM)] or [^64^Cu]CuCl_2_, as therapeutic agents, in addition to imaging tumor hypoxia with PET. The ability of copper-64 for molecular radiotherapy is based on its complex decay scheme, which includes β^+^ (0.656 MeV, 19%), β^−^ (0.573 MeV, 40%), electron capture (41%), and gamma emission (1.346 MeV, 0.5%), with a half-life of 12.7 h [42]. In particular, electron capture will generate a cascade of Auger electrons with high linear energy transfer (LET) in tissue, which has been demonstrated to induce tumor cell death with high efficiency due to the release of the electrons in close proximity to the DNA [43, 44]. Auger electrons from copper-64 are considered high LET radiation with ~ 2 keV of average energy and with ~ 126 nm average range in tissue, which is also a desirable property as these electrons travel much shorter than the diameter of tumor cells compared to other common β^−^ emission radionuclide therapies where the electrons from β^−^ emissions have much longer range [45–47].

The effectiveness of copper-64 as a therapeutic agent has been experimentally demonstrated in a number of in vitro and in vivo studies with various solid tumors. As early as in 2001, Lewis et al. demonstrated in a human GW39 colon cancer model in hamster that the survival rate of hamsters administrated with [^64^Cu][Cu(ATSM)] was significantly increased compared to untreated hamsters, with no acute toxicity even at high administered doses (10 mCi, 370 MBq) [46]. Subsequently, [^64^Cu][Cu(ATSM)] was proposed as a potential molecular radiotherapy agent [46]. An early in vitro investigation by Obata et al. characterized the molecular basis of [^64^Cu][Cu(ATSM)] as a therapeutic agent in a mouse Lewis lung carcinoma LL/2 model and showed that [^64^Cu][Cu(ATSM)] reduced the survival rate of clonogenic tumor cells in a dose-dependent manner, in which uptake of 1.50 Bq/cell resulted in a 99% killing rate [48]. The study further confirmed with the alkali comet assay that significant DNA damage was observed in cells exposed to [^64^Cu][Cu(ATSM)]. Combined with the lack of increase of apoptotic and necrotic cells, the damage was considered to be direct intracellular radiation damage [48]. These authors also hypothesized that the electrons from β^−^ emission contributed to tumor cell killing, since the majority of the intracellular [^64^Cu][Cu(ATSM)] was found to remain in the post-mitochondrial supernatant [48]. However, other studies have demonstrated that Auger electrons are cytotoxic even without internalization, due to nuclides remaining on the cell membrane [49, 50]. Correlative studies using [^64^Cu][Cu(ATSM)], [^18^F]-FDG, and immunohistochemical staining showed that [^64^Cu][Cu(ATSM)] mainly accumulates at the edges of the tumor outside of [^18^F]-FDG accumulated regions, where the colony-forming ability is significantly higher and is considered quiescent but clonogenic [19]. Using a radioresistant hypoxic MCF-7 breast cancer model, Weeks et al. found that the increased uptake of [^64^Cu][Cu(ATSM)] in hypoxic MCF-7 tumor cells can effectively induce sufficient damage to the DNA of clonogenic tumor cells [51]. At reduced atmospheric oxygen concentration, uptake of [^64^Cu][Cu(ATSM)] in MCF-7 tumor cells was shown to be significantly increased, and for the first time, a strong correlation between the expression of HIF-1α and [^64^Cu][Cu(ATSM)] uptake by tumor cells was demonstrated [51].

Using a mouse colon carcinoma model, Yoshii et al. further demonstrated that [^64^Cu][Cu(ATSM)] preferentially accumulated in regions with high CD133^+^ expression, a common marker of CSC, which is believed to be the origin of self-renewal and differentiation ability of many tumor types and a major contributor to therapeutic resistance and metastasis [20, 52–56]. Expression of CD133^+^ increased with increasing hypoxia, where the uptake of [^64^Cu][Cu(ATSM)] also increased [20]. Following this in vivo mechanistic study, it was shown in a therapeutic study that administration of 37 MBq [^64^Cu][Cu(ATSM)] inhibited tumor growth, decreased the CD133^+^ cell percentage, and the ability to metastasize [57]. If [^64^Cu][Cu(ATSM)] indeed accumulates in regions of tumor hypoxia, and preferentially, regions of CSCs or stem-like cells, as previously reported based on cancer cell expression of CD133^+^, then it would be an excellent therapeutic agent not only for hypoxic tumors but also for targeting CSCs.

Lately, more clinical and preclinical studies suggested a therapeutic effect of the copper-64 radiopharmaceuticals [^64^Cu][Cu(ATSM)]/[^64^Cu]CuCl_2_ in targeting hypoxic, aggressive solid tumors. In a clinical investigation involving 50 human prostate cancer patients, Piccardo et al. and Righi et al. reported that [^64^Cu]CuCl_2_ is effective in detecting local recurrence and lymph node metastasis, although the evidence is still limited and further clinical trials are necessary to obtain more conclusive results [37, 58]. Glioblastoma multiforme (GBM), the most common and aggressive primary brain tumor in adults which typically is associated with poor prognosis, is known to have hypoxic regions that are resistant to chemo- and radiotherapy, as well as frequent recurrences after surgery [59–61]. Using a mouse xenograft GBM model, Ferrari et al. reported that intravenous administration of 333 MBq [^64^Cu]CuCl_2_ resulted in significant tumor reduction, ranging from 68–94% (single dose (SD), two complete tumor disappearance cases) and 64–94% (multiple dose (MD), 55.5 MBq × 6, four complete tumor disappearance cases), with subsequent prolonged survival rate at 73.3% (SD) and 73.0% (MD) at the 20th week, whereas in the control group, all tumors increased in the first 8 weeks [29]. It was found that exposure of [^64^Cu]CuCl_2_ inhibits the neurosphere-forming ability of tumor cells, a prerequisite for the formation of tumor masses [29]. Similar therapeutic effects has also been demonstrated with [^64^Cu][Cu(ATSM)] in a parallel preclinical study by Yoshii et al. Intravenous administration of [^64^Cu][Cu(ATSM)] in single doses (up to 148 MBq) and multiple doses (37 MBq × 4) has resulted in various degrees of tumor volume reduction in dose-dependent manners. The most successful tumor reduction and prolonged survival was observed with multiple doses of [^64^Cu][Cu(ATSM)] (37 MBq × 4), in which 86% (six out of seven) of cases achieved near complete tumor disappearance [62]. To further evaluate the possibility of using [^64^Cu][Cu(ATSM)] for internal radiotherapy, Matsumoto et al. investigated the pharmacokinetics and safety aspects of stable [Cu(ATSM)] (stable copper is a mix of ^63^Cu and ^65^Cu) and free H_2_-ATSM in a preclinical mouse study. Therapeutic administration of stable [Cu(ATSM)] and H_2_-ATSM in a ratio of 2:25 was found to be safe for patients when given as 15 μg once per week for 4 weeks [63]. In addition, [^64^Cu][Cu(ATSM)] therapy have been shown to be a novel approach to enhance cancer treatment efficiency concomitantly with bevacizumab in a human colon carcinoma xenograft model, where the repeated use of bevacizumab has caused decreased tumor vascularization and tumor hypoxia. Administration of [^64^Cu][Cu(ATSM)] in addition to bevacizumab treatment has shown to reduce tumor size and lead to prolonged survival, without major adverse effects [64].

A list of preclinical investigations on the therapeutic effects of copper-64 is summarized in Table 2. These studies have repeatedly demonstrated that [^64^Cu][Cu(ATSM)]/[^64^Cu]CuCl_2_ can be used not only as PET imaging tracers, but simultaneously by increasing the administrated dose to effectively target tumor cells, and perhaps the CSCs [19, 57], thereby significantly inhibit tumor growth with few adverse effects. The optimal administration method is likely to use multiple smaller doses to reduce possible adverse effects while keeping sustained internal radiation DNA damages of hypoxic tumor cells [29, 62]. Though the copper retention mechanism remains not fully understood, the trapping of copper-64 in hypoxic regions combined with the decay scheme of copper-64 with high LET, short-range Auger electrons clearly contributes to such therapeutic effects. In addition to be a stand-alone therapeutic agent, copper-64 radiopharmaceuticals have also been shown to address tumor hypoxia in combinatory cancer treatments (e.g., bevacizumab [64]), which opens new avenues of possibilities.

**Table 2:** Preclinical studies using copper-64 as therapeutic agent

Tracer	Tumor type	Results/Conclusions	Reference
[^64^Cu][Cu(ATSM)]	Colon cancer	High dose (370 MBq) of [^64^Cu][Cu(ATSM)] significantly increased the survival rate for tumor bearing animals with no acute adverse effects.	[46]
[^64^Cu][Cu(ATSM)]	Lung cancer	[^64^Cu][Cu(ATSM)] highly accumulated in hypoxic region of the tumor and reduced the survival rate of clonogenic tumor cells in a dose-dependent manner, with observed DNA damage from copper-64 radiation.	[48]
[^64^Cu][Cu(ATSM)]	Lung cancer	Cells in high [^64^Cu][Cu(ATSM)] regions were quiescent but highly clonogenic under mild hypoxia. These cells should be attacked by either [^64^Cu][Cu(ATSM)] internal radiotherapy and/or other intensive-modulated radiotherapy.	[19]
[^64^Cu][Cu(ATSM)]	Breast cancer (MCF-7)	Uptake of [^64^Cu][Cu(ATSM)] as well as radio toxicity increased with decreased atmospheric oxygen level. Statistically significant increase of [^64^Cu][Cu(ATSM)] DNA damage in hypoxia confirmed by Comet assay. Hypoxia-enhanced uptake of copper-64 may be used to overcome radio-resistance of hypoxic MCF-7 cells.	[51]
[^64^Cu][Cu(ATSM)]	Colon cancer	[^64^Cu][Cu(ATSM)] treatment inhibited tumor growth and reduced tumor volume. Percentage of CD133^+^ cancer stem cells and metastatic ability in treated cells were decreased.	[57]
[^64^Cu]CuCl_2_	Brain cancer (GBM)	Treatment with [^64^Cu]CuCl_2_ inhibited the neurosphere-forming ability of GBM cell lines. A therapeutic dose led to significant decreased of complete regression of tumor volume, followed by prolonged survival with no significant adverse effects observed.	[29]
[^64^Cu][Cu(ATSM)]	Colon cancer	[^64^Cu][Cu(ATSM)] can be used to address the effects of tumor hypoxia and increased HIF-1 activation caused by repeated use of bevacizumab, which has been shown to inhibit tumor growth and prolong survival in bevacizumab-treated mice.	[64]
[^64^Cu][Cu(ATSM)]	Brain cancer (GBM)	Single administration of high-dose (148 MBq) [^64^Cu][Cu(ATSM)] inhibited tumor growth and prolonged survival in a dose-dependent manner, with slight adverse effects. Multiple administration (4 × 37 MBq) showed optimal results for tumor inhibition and survival, with no observed adverse effects.	[62]
[^64^Cu][Cu(ATSM)]/[^64^Cu]CuCl_2_		[^64^Cu][Cu(ATSM)] and [^64^Cu]CuCl_2_ shown similar in vivo accumulation in the hypoxic regions of the tumor, while [^64^Cu][Cu(ATSM)] also stained non-hypoxic regions with high expression of copper transporters. In vitro [^64^Cu][Cu(ATSM)] has siginificant higher accumulation than [^64^Cu]CuCl_2_.	[68]

### Controversial copper uptake mechanism

Although the effectiveness of using [^64^Cu][Cu(ATSM)] for the imaging of tumor hypoxia with PET has been repeatedly demonstrated, the copper uptake mechanism is still controversial and may explain the limited clinical use. Conventionally, as proposed initially by Fujibayashi et al. in 1997, Cu(II)-[Cu(ATSM)] was thought to diffuse into tumors due to its high membrane permeability, where Cu(II)-[^62^Cu][Cu(ATSM)] was reduced to Cu(I)-[^62^Cu][Cu(ATSM)] by NADH/NADPH in the dysfunctional mitochondria (due to hypoxia) with disturbed electron flow [13]. Cu(I)-[Cu(ATSM)] is much less stable than Cu(II)-[Cu(ATSM)], resulting in dissociation of copper from the complex, forming H_2_-ATSM which thus results in the retention of radiocopper. A mechanistic study revealed that unlike brain cells, the retention of stable [Cu(ATSM)] in tumor cells was mostly found in the microsome/cytosol fraction and not in the mitochondrial fraction. It was suggested that the reduction of copper is caused by microsomal and cytosolic enzymes including NADH and NADPH [65]. A recent review by Colombié et al. discussed various controversial aspects of [^64^Cu][Cu(ATSM)] for imaging tumor hypoxia, concluding that [^64^Cu][Cu(ATSM)] is more than a hypoxia tracer; its accumulation in tumor cells is also linked to the redox potential and reactive oxygen species which reflects the overreduced cellular state [66]. Although the exact reduction mechanism of copper in the tumor is debated, the foundation of the ligand-based dissociation mechanism is that the Cu(II)-[Cu(ATSM)] complex remains intact after the administration in the blood plasma, until encountering a hypoxic region of the tumor where the Cu(II) can be irreversibly reduced to Cu(I), and subsequently be trapped.

However, studies using ionic copper-64 have raised questions on the in vivo retention mechanism. As mentioned, a direct in vitro and in vivo comparison between [^64^Cu][Cu(ATSM)] and [^64^Cu]Cu-acetate has showed essentially mirroring in vivo results of the copper-64 oxygen-dependent uptake and biodistribution, while the in vitro results showed significantly less hypoxia selectivity of [^64^Cu]Cu-acetate [28]. The same study also reported concerning stability issues of the complexing ligand in mouse blood serum and whole blood in vivo, despite being stable in mouse serum in vitro. Similarly, comparing two recent studies investigating the theranostic potentials of copper-64 with glioblastoma in similar setups but by two different groups, showed surprisingly similar results for [^64^Cu]CuCl_2_ study and [^64^Cu][Cu(ATSM)], having near identical dose-dependent survival rates [29, 62]. Furthermore, a recent pilot study using Cu(I) for PET imaging of experimental melanoma models demonstrated that the in vivo cellular uptake and biodistribution between [^64^Cu]CuCl_2_ and [^64^Cu]CuCl are near identical in B16F10 and A375 tumors in mice, despite significant difference in in vitro cellular uptake, providing evidence to further question the conventional explanation of the copper trapping mechanism of [^*^Cu][Cu(ATSM)] [67]. Pérès et al. recently reported a direct comparison between [^64^Cu][Cu(ATSM)] and [^64^Cu]CuCl_2_ both in vivo and in vitro in the glioblastoma C6 model [68]. In vivo, both [^64^Cu][Cu(ATSM)] and [^64^Cu]CuCl_2_ showed significantly elevated accumulation in the hypoxic regions as confirmed by overlapping ex vivo autoradiographs with [^18^F]F-MISO and pimonidazole staining, but where [^64^Cu][Cu(ATSM)] also stained some non-hypoxic regions with high expression of copper transporters [68]. [^64^Cu][Cu(ATSM)] showed greater uptake in both healthy brain and malignant tumors than [^64^Cu]CuCl_2_, while [^64^Cu]CuCl_2_ showed a greater tumor/brain ratio [68]. In vitro, elevated accumulation of [^64^Cu][Cu(ATSM)] was detected in severe hypoxia (pO_2_ < 0.5%), which further increased with decreasing oxygen concentration. Accumulation of [^64^Cu]CuCl_2_ was elevated at pO_2_ < 5%, but with no further significant increase at lower oxygen concentrations [68]. The authors concluded that while both [^64^Cu][Cu(ATSM)] and [^64^Cu]CuCl_2_ could be considered as hypoxia-selective tracers, non-hypoxic accumulation in copper transporters should also be accounted for [68]. Interestingly, in an investigation of the possibility to use [^64^Cu][Cu(ATSM)] and [^64^Cu][Cu(PTSM)] as substrates for imaging the multidrug resistant type 1 (MDR1) protein in predicting chemotherapy response, Liu et al. demonstrated in liver tumor mouse models that expression of MDR1 decreased the retention of [^64^Cu][Cu(ATSM)] and [^64^Cu][Cu(PTSM)] with enhanced efflux, while knockdown of MDR1 showed inverse effects, which further implies a more complex copper retention mechanism than what was previously proposed [69].

These preclinical results are critical for the understanding of clinical studies using ionic radiocopper salts for PET imaging of tumors, in which the correlation between tumor oxygenation and the radiocopper uptake is much more difficult to address directly. The direct comparisons in preclinical studies have challenged the conventional understanding of the copper uptake mechanism in tumors, in which the role of copper-64 itself in the copper metabolism pathway should be addressed and the function of the ligand is in question due to its potential lack of stability in blood. In fact, an increased number of clinical studies with copper-64-based tracers for PET imaging applications have in recent years used [^64^Cu]CuCl_2_, and not [^64^Cu][Cu(ATSM)] (Table 1). To better understand the copper uptake mechanism, it is necessary to revisit the role of copper and its transportation in mammals.

### Copper metabolism

**Fig. 1:**
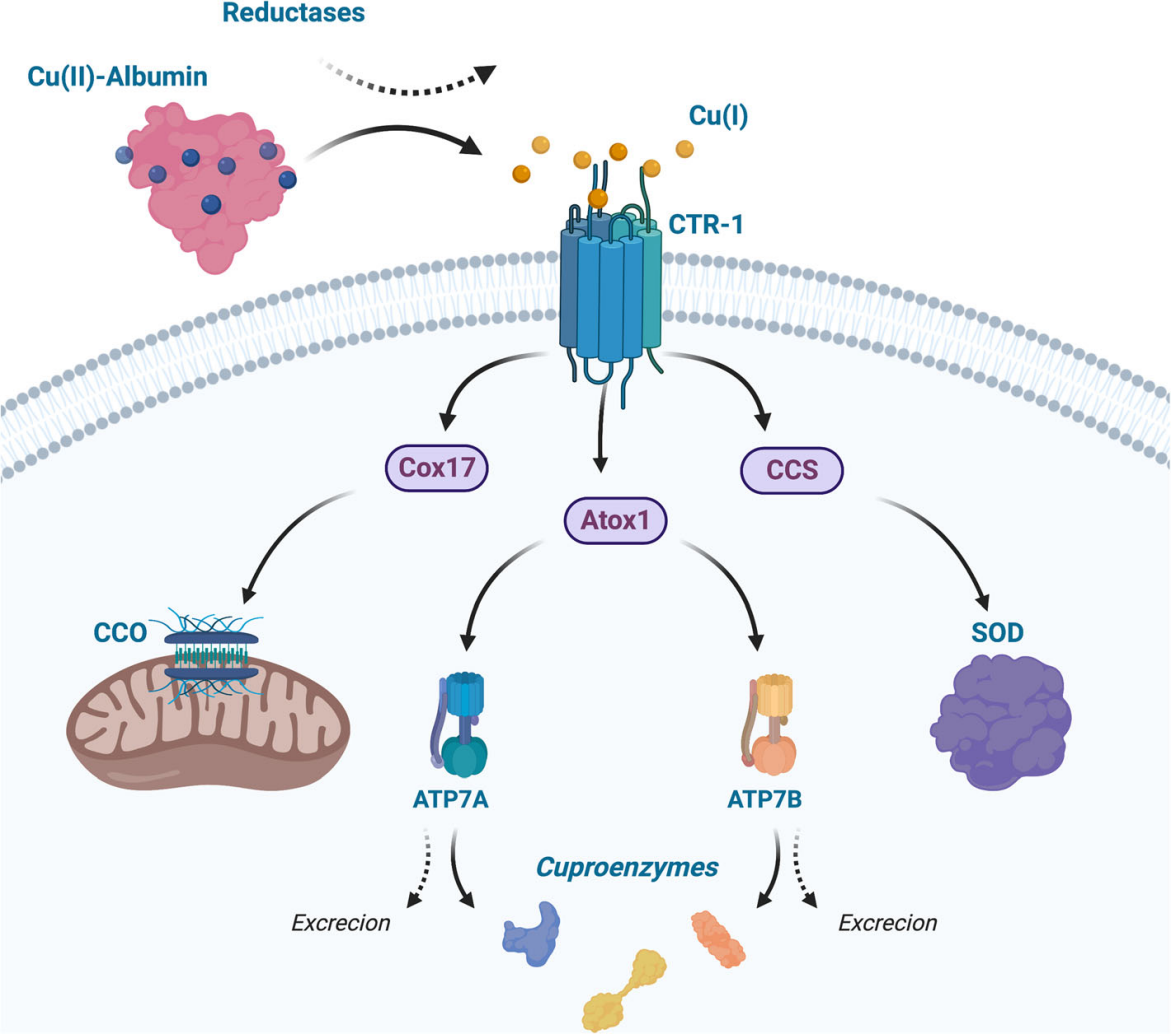
Simplified illustration of copper transport mechanism. Cu(II) is carried by albumin or transcuprein in blood, and is believed to be reduced to Cu(I) by reductases near the surface of cells. Copper transporter 1 (CTR-1) is the major high-affinity copper transporter, which transports Cu(I) into the cells and deliver to the copper chaperone Cox17, the antioxidant protein 1 (Atox1), or the copper chaperone for superoxide dismutase (CCS). Atox1 further deliver copper to either the copper transporting adenosine triphosphatase (ATPase) ATP7A in neuron/astrocyte cells, or the ATPase ATP7B in the liver, which either pass the copper to target cuproenzymes, or to excretion

Copper is an essential element in mammals and is required as a cofactor for a number of cuproenzymes, mainly distributed in liver, muscle, bond, and blood [70]. Copper uptake and distribution is regulated by proteins including CTR-1, copper transporter 2 (CTR-2), copper chaperones such as antioxidant protein 1 (Atox1), copper chaperone for superoxide dismutase (CCS), cytochrome c oxidase copper chaperone 17 (Cox17), and the copper transporting adenosine triphosphatases (ATPases) ATP7A and ATP7B, as shown in Fig. 1 [71–73]. Free copper ions are typically absent in blood or cytoplasm, since any presence of ionic copper in either Cu(I) or Cu(II) will rapidly associate with three known high-affinity protein carriers including albumin, transcuprein, and ceruloplasmin [70, 74, 75]. Uptake of copper from blood is mainly through the high-affinity CTR-1, which transports copper in the form of Cu(I) into the cells as bioavailable copper in the cytosol [76–78]. Explicit evidence has also shown that copper in blood plasma is carried by proteins but not low molecular weight amino acids or complexes [70, 79]. It has been demonstrated that transport of copper from blood uptake contains two phases. In the first phase after injection, copper will be absorbed rapidly by albumin (which contains high-affinity copper(II) binding sites [74, 75]) and transcuprein, and again exclusively bind to proteins but not low molecular weight complexes or as free form, reaching a minimum level in plasma within approximately 2 h [80, 81]. In this phase, the main destinations of copper are the liver (~ 40%), muscles (~ 18%), and the kidneys (~ 6%) [80]. Reemergence of copper in plasma starts from 6 h to approximately 1 day after initial injection at which point the blood copper concentration reaches another maximum, this time incorporated with ceruloplasmin and being transported to other tissues [80].

To transport copper into the cells, albumin or transcuprein delivers copper to the extracellular domain of the plasma membrane transporter CTR-1, which then imports copper across the cell membrane and further deliver to copper chaperone proteins [82, 83]. Copper(II) is believed to be reduced to Cu(I) during this stage by metalloreductases, but the exact reduction process and maintenance of Cu(I) with the presence of oxygen before reaching CTR-1 remains unclear [76]. Copper chaperone protein Atox1 delivers Cu(I) to ATP7A (neuron/astrocyte cells) or ATP7B (liver), before it is delivered to the target cuproenzymes, such as cytochrome c oxidase (CCO) in mitochondrial via Cox17 or copper/zinc-superoxide dismutase via CCS [83].

In a copper transport perspective, regulation of CTR-1 is crucial, and in a hypoxic environment, the HIFs mediate the signaling cascade and trigger a series of cellular responses in both normal and cancer tissues [84]. HIFs respond to hypoxia by altering the gene expression involving at least 150 proteins that control critical cellular functions such as metabolism and survival [84]. Under normoxia, HIF-1α is regularly produced and degraded, achieving an equilibrium; under hypoxia, HIF-1α is stabilized, activating transcriptions in combination with other coactivators, including factors that promote the self-renewal capability, multipotency, adaptivity, as well as inhibition of differentiation of CSCs, in effect giving CSCs survival advantages and enhanced self-renewed proliferation [20, 22, 84]. Notably, copper is required for the activation of HIF-1 via HIF-1α binding to the hypoxia-responsive element and the formation of the HIF-1 transcriptional complex [85, 86]. As a result, these studies predict a favorable environment for CSCs and its self-renewing proliferation under hypoxia with the activation of HIF-1, leading to an overall worsened cancer prognosis as well as potential resistance to chemoradiotherapy. Indeed, such correlation has been widely observed and reported in various cancers, including ovarian cancer [87], cervical cancer [88, 89], breast cancer [90], lung cancer [91], bladder cancer [92], and glioma [93].

Similarly, hypoxia has been shown to stimulate the expression of CTR-1 [94], and upregulation of CTR-1 has also been repeatedly reported in hypoxic tumors [25, 29, 95–99]. Peng et al. reported a significantly higher [^64^Cu]CuCl_2_ uptake in tumor tissue compared to normal tissue, meanwhile observing a substantial increase of CTR-1 expression in tumor but not in normal tissue [25]. Cai et al. provided in vitro evidence with [^64^Cu]CuCl_2_ that by knocking down CTR-1 in tumors, showing that the uptake of [^64^Cu]CuCl_2_ was reduced compared to the control group, along with the suppression of tumor cell proliferation [96]. Qin et al. showed high, specific uptake of [^64^Cu]CuCl_2_ accompanied by overexpression of CTR-1 in melanoma cell lines [97]. Remarkably, CTR-1 transports copper in the form of Cu(I) instead of copper(II) [76–78], thus, if the upregulation of CTR-1 indeed is responsible for the transport of copper to the hypoxic tumor sites, copper(II) must have been disassociated and reduced to Cu(I) in blood, before it is delivered to the tumor through the copper secretory pathway.

### Dosimetry

One important aspect to consider when using copper-64-based radiopharmaceuticals for PET diagnostics, and especially for therapeutic applications, is radiation dosimetry. However, such evaluation is difficult due to the complexity of the copper-64 decay scheme, especially when considering absorbed dose from high-LET Auger electron emissions. An early study on the therapeutic effect of [^64^Cu][Cu-(ATSM)] by Lewis et al. suggested that the absorbed dose from Auger electrons is dependent on the distance between copper-64 ions and the cell nucleus, since the Auger electrons have low energy and very short range in tissue but can induce significant DNA damage in close proximity to the cell nucleus [46]. In an analysis of [^60/61/62/64^Cu][Cu(ATSM)] by Laforest et al. [100], the biodistribution was measured in five patients using [^60^Cu][Cu(ATSM)] and the organ doses were calculated according to the Medical Internal Radionuclide Dose (MIRD) method. It was estimated that for [^64^Cu][Cu(ATSM)] the effective dose was 0.036 mSv/MBq, while the highest organ dose was in the liver with an absorbed dose of 0.390 mGy/MBq [100]. Similarly, in a clinical investigation of [^64^Cu]CuCl_2_ PET/CT in prostate cancer staging, Capasso et al. reported that liver is the organ with the highest absorbed dose, with an estimated dose of 0.294 mGy/MBq, approximately ten-fold of the estimated full body dose of 0.0266 mGy/MBq [36]. Concerning that the dosimetry evaluation by Capasso et al. contained limited data points and was not the main focus of the investigation, Avila-Rodriguez et al. evaluated [^64^Cu]CuCl_2_ biodistribution and dosimetry in healthy human beings. They estimated that the effective dose was 0.0512 mSv/MBq for men and 0.0618 mSv/MBq for women, while the absorbed dose in the liver was 0.310 mGy/MBq (men) and 0.421 mGy/MBq (women) [101]. For a more comprehensive list of dosimetry estimations in different organs, we refer readers to the original article in reference [101].

A similar dosimetry estimation was also reported by Panichelli et al. in a clinical study of brain tumors involving 19 patients with [^64^Cu]CuCl_2_ PET. The highest dose-absorbing organ was the liver, with an absorbed dose of 0.321 mGy/MBq, while the full body dose was estimated to be 0.0312 mGy/MBq [38]. In a recent clinical study using [^64^Cu]CuCl_2_ to detect prostate cancer relapse involving 50 patients, Piccardo et al. evaluated the absorbed dose to each organ. The authors reiterated that the critical organ is the liver, as reported by prior study [36], and found that the effective dose for [^64^Cu]CuCl_2_ was 0.0283 mSv/MBq, comparable to that of ^18^F-choline (0.02 mSv/MBq) and [^68^Ga][Ga-(PSMA)] (0.0236 mSv/MBq) [37]. However, it is worth noting that the absorbed dose for the liver is considerably higher in [^64^Cu]CuCl_2_ PET with 0.271 mGy/MBq, compared to ^18^F-choline (0.0610 mGy/MBq) and ^68^Ga-PSMA (0.0309 mGy/MBq) [37]. The authors argued that the increased radiation exposure is negligible, although the effects to liver was not specifically discussed [37].

Despite evaluation in several preclinical and clinical studies, the accurate dosimetry of copper-64 to tumor and organs remains yet to be established. As previously mentioned, a detailed and accurate dosimetry description is challenging because of the short-range high-LET Auger electron emission, which is believed to be more radiotoxic than β^−^/β^+^ emission and being the main contributor of the therapeutic effect. However, the distance-dependent dose deposit nature complicated the evaluation, since the degree of internalization of copper-64 ions must be taken into account.

## Discussion

### Reconsidering the copper pathways under tumor hypoxia

**Fig. 2:**
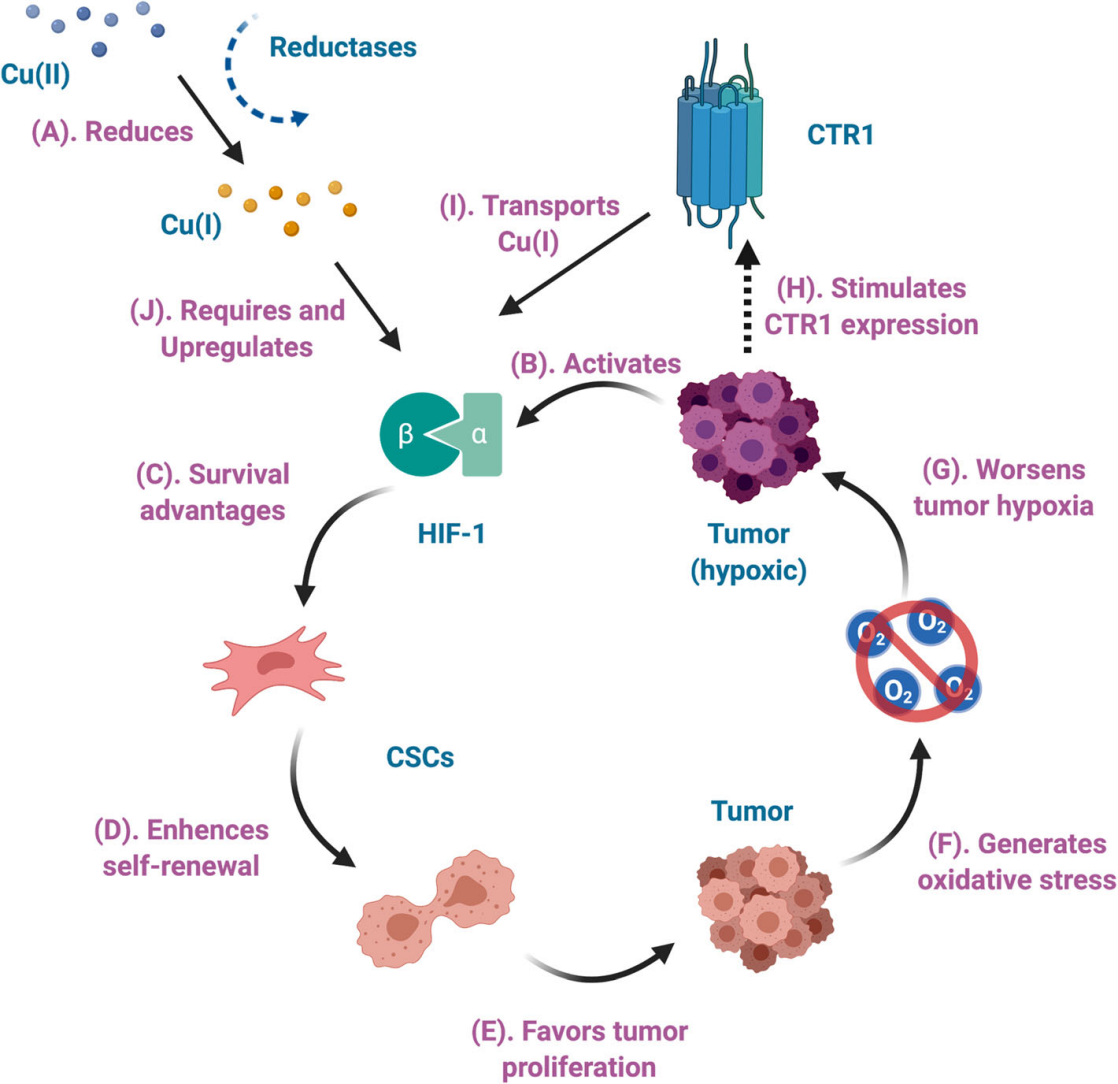
Possible role of copper in tumor hypoxia. **a** Cu(II) is reduced to Cu(I) by metalloreductases. **b** Hypoxic tumor activates the hypoxia-inducible factor 1 (HIF-1). **c** Activation of HIF-1 promotes cancer stem cells (CSCs) via survival advantages, which **d** enhances the self-renewal ability and inhibitsdifferentiation of CSCs. **e** In effect this is in favor of tumor proliferation, which **f** generates oxidative stress, and **g** further worsen tumor hypoxia. On the other hand, **h** tumor hypoxia stimulates copper transporter 1 (CTR-1) expression, increases the production of CTR-1 to **i** transport incoming Cu(I), **j** upregulating the HIF-1 expression, where Cu(I) is required

Based on our review of copper-64 radiopharmaceuticals in PET imaging and therapeutic purposes, we propose an emphasized role of copper itself in the copper uptake in hypoxic tumors, as shown in Fig. 2. Starting with ionic copper(II) in blood, either from direct injection of ionic copper compounds or through dismantling of copper(II) complexes, copper(II) is quickly reduced to Cu(I) by reductases [102–105], immediately binding to copper-binding proteins. On the other hand, tumor hypoxia stimulates CTR-1 expression [29, 94], which then binds to Cu(I) and is transported into the tumor cells. With Cu(I) available, hypoxic tumor cells activates HIF-1 [22, 23, 84, 87], triggering a series of effects including promotion of CD133^+^ CSCs via survival advantages [20, 22, 23, 57, 84, 87, 93], which also enhances the self-renewal ability and inhibits differentiation of CSCs [22, 84, 106]. In effect, this increases the tumor proliferation and generates oxidative stress, resulting in worsened tumor hypoxia [19, 36, 84, 107–109]. In the meantime, this picture can also help us to understand the efficiency of copper-64 radiopharmaceuticals in preclinical molecular radiotherapy studies. Clonogenic hypoxic tumors activate HIF-1 and have elevated CTR-1 expression, thus significantly increasing the uptake of copper-64. Localized high concentration of copper-64 emits high-LET Auger electrons at close proximity of these proliferating cancer cells, causing substantial DNA damages and eventually lead to significant tumor reduction.

## Conclusions

Detecting and quantifying tumor hypoxia is one of the most critical yet challenging tasks for cancer management. In this review, we have summarized preclinical and clinical evidence that support the possibility of using both [^64^Cu][Cu(ATSM)] and ionic ^64^Cu(II) salts for imaging of tumor hypoxia using PET. These evidences support the possibility of using copper-64-PET to detect and quantify hypoxia in solid tumors in clinical practice and thereby enable the potential to provide more individualized and optimized cancer treatment. In addition, the possibility to use copper-64 as a therapeutic agent provides a promising approach to target clonogenic cancer cells, cancer stem cells, and stem-like cells that are challenging in conventional treatment, and which may contribute to improved patient outcomes. Together, copper-64 radiopharmaceuticals [^*^Cu][Cu(ATSM)]/[^*^Cu]CuCl_2_ have the potential to serve as theranostic agents that can simultaneously provide both diagnostics and treatment for cancer patients.

On the other hand, the current understanding of the copper retention mechanism remains to be fully resolved before using copper for therapeutic purposes in humans. Emerging evidence has challenged the understanding of the metal-ligand dissociation mechanism of Cu(II)-[Cu(ATSM)], specifically questioning the validity of the assumption that the complex can stay intact in blood. Several studies have also addressed the importance to understand the role of copper itself in the retention, although the exact pathways remain unclear and requires further investigations. In summary, the emerging preclinical and clinical studies as well as potential clinical applications of copper-64 radiopharmaceuticals in PET imaging and theranostics would benefit from further mechanistic investigations in order to identify the exact copper retention mechanism.

## Data Availability

Data sharing not applicable to this article as no datasets were generated or analyzed during the current study.

## Abbreviations

[Cu(ATSM)]: Copper(II)-diacetyl-bis(N4-methylthiosemicarbazone)
Cu(II)-[Cu(ATSM)]: Copper(II)-diacetyl-bis(N4-methylthiosemicarbazone), with emphasis on Cu(II)
Cu(I)-[Cu(ATSM)]: Copper(I)-diacetyl-bis(N4-methylthiosemicarbazone), with emphasis on Cu(I)
[Cu(PTSM)]: Copper(II)-pyruvaldehyde-bis(N4-methylthiosemicarbazone)
CSCs: Cancer stem cells
CTR-1: Copper transporter 1
CTR-2: Copper transporter 2
*Cu: Radioactive (β+) copper isotopes, “*” may be replaced by atomic mass numbers
EPR: Electron paramagnetic resonance
[^18^F]-FAZA: [^18^F]-Fluoroazomycin arabinoside
[^18^F]-FDG: [^18^F]-Fluorodeoxyglucose
[^18^F]-FMISO: [^18^F]-Fluoromisonidazole
fMRI: Functional magnetic resonance imaging
GBM: Glioblastoma
HIF-1: Hypoxia inducible factor 1
HIF-1α: Hypoxia inducible factor 1, alpha subunit
HRE: Hypoxia response element
LET: Linear energy transfer
LLC: Lewis lung carcinoma cell line
PET: Positron emission tomography
PSMA: Prostate specific membrane antigen
SD/MD: Single dose/multiple dose
